# Effectiveness and safety of adalimumab in patients with ankylosing spondylitis or psoriatic arthritis and history of anti-tumor necrosis factor therapy

**DOI:** 10.1186/ar3054

**Published:** 2010-06-16

**Authors:** Martin Rudwaleit, Filip Van den Bosch, Martina Kron, Sonja Kary, Hartmut Kupper

**Affiliations:** 1 Medical Department I, Rheumatology, Charité, Campus Benjamin Franklin Hospital, Hindenburgdamm 30, 12200 Berlin, Germany; 2 Department of Rheumatology, University Hospital Gent, De Pintelaan 185, B-9000 Gent, Belgium; 3 Abbott GmbH & Co. KG, Knollstrasse 50, Ludwigshafen, 67061, Germany

## Abstract

**Introduction:**

Tumor necrosis factor (TNF) antagonists reduce the signs and symptoms of spondyloarthritides, including ankylosing spondylitis (AS) and psoriatic arthritis (PsA). Our objective was to evaluate the effectiveness and safety of adalimumab, 40 mg every other week, for patients with AS or PsA and prior treatment with infliximab (IFX) and/or etanercept (ETN).

**Methods:**

Both trials were 12-week, open-label studies with an optional extension period up to week 20. Patients were stratified by history of anti-TNF treatment, prior anti-TNF therapy received (IFX, ETN, or both), and reason for discontinuation of prior TNF antagonist. ETN was discontinued ≥ 3 weeks, and IFX was discontinued ≥ 2 months before the first adalimumab administration. Effectiveness at week 12 was evaluated by using observed standard-outcome measurements for AS and PsA.

**Results:**

At week 12 of adalimumab treatment, Bath Ankylosing Spondylitis Disease Activity Index 50 responses were achieved by 40.8% of 326 patients with AS who had received prior anti-TNF therapy and by 63.0% of 924 patients with AS who were naive to TNF antagonist. Observed response rates were generally greater for patients who discontinued the prior anti-TNF therapy because of loss of response or intolerance than for patients who discontinued because of lack of response. Median changes in swollen-joint count and in enthesitis score were similar in patients with and without prior TNF-antagonist treatment. Modified PsA response criteria were fulfilled by 71.2% of 66 patients with PsA, with prior exposure to TNF antagonists, and by 78.8% of 376 patients with no history of anti-TNF therapy. The percentages of patients with PsA attaining a Physician's Global Assessment of psoriasis of "Clear/Almost clear" increased from 33.3% to 61.0% for patients with prior IFX and/or ETN treatment and from 34.6% to 69.7% for patients without anti-TNF therapy. The median change in the Nail Psoriasis Severity Index was -6 for both groups. In both studies, patterns of adverse events were similar for patients with and without prior anti-TNF therapy and were consistent with the known safety profile of adalimumab.

**Conclusions:**

Patients with AS or PsA previously treated with IFX and/or ETN experienced clinically relevant improvements of their diseases after 12 weeks of adalimumab.

**Trial registrations:**

ClinicalTrials.gov NCT00478660 and NCT00235885.

## Introduction

Agents that target tumor necrosis factor (TNF) are highly effective in treating patients with active rheumatic disorders, such as rheumatoid arthritis (RA), ankylosing spondylitis (AS), or psoriatic arthritis (PsA) [1]. Nevertheless, patients may not respond optimally to or may be intolerant of treatment with a given TNF antagonist. A practical question faced by clinicians and patients is whether switching to another TNF antagonist is likely to result in an improved therapeutic response.

Treatment with a second or third TNF antagonist has been shown to be successful and well tolerated in a substantial percentage of patients with RA, regardless of the order of subsequent therapies (etanercept (ETN), infliximab (IFX), or adalimumab) [2-6]. In RA, a patient's failure to respond to one TNF antagonist does not predict failure with a second anti-TNF agent [6-9], and it is rare for a patient to fail to respond to three [10]. However, analyses of switching to another TNF antagonist for patients with spondyloarthritides, such as AS or PsA, are quite limited and often represent a minor subgroup of patients with various rheumatic diseases evaluated in national registries [2,3,11-16].

Adalimumab, a fully human monoclonal antibody that binds to and neutralizes TNF, is approved for the treatment of AS, PsA, RA, psoriasis, juvenile idiopathic arthritis, and Crohn disease in Europe, Canada, the United States, and other world regions [17]. In two open-label clinical studies, we investigated the effectiveness and safety of adalimumab in treating patients with active AS or PsA who had a history of therapy with IFX or ETN or both: Review of Safety and Effectiveness witH Adalimumab in Patients with Active Ankylosing SpOnDYlitis (RHAPSODY) and SafeTy and Efficacy of Adalimumab in Patients with Active Psoriatic Arthritis (PsA): An Open-Label, Multinational Study to Evaluate the Response to Every-Other-Week Adalimumab When Added to Insufficient Standard Therapy including Patients Who Failed Prior Treatment With Other TNF-Inhibitors (STEREO) [18,19]. These analyses included stratification by prior anti-TNF treatment received (IFX, ETN, or both) and by the reason for discontinuation of the prior anti-TNF therapy.

## Materials and methods

### Patients

Adults at least 18 years of age with AS according to the 1984 modified New York criteria for AS [20] for at least 3 months and a Bath Ankylosing Spondylitis Disease Activity Index (BASDAI) [21] score ≥ 4 and failure of ≥ 1 nonsteroidal antiinflammatory drugs (NSAIDs) were eligible to enroll in RHAPSODY [18]. The STEREO study enrolled adults at least 18 years of age with PsA diagnosed by a rheumatologist, three or more swollen and three or more tender joints, and failure of one or more disease-modifying antirheumatic drugs (DMARDs) [19]. Prior treatment with ETN and with IFX was allowed in both studies if ETN was discontinued ≥ 3 weeks and IFX was discontinued ≥ 2 months before the first adalimumab injection [18,19].

### Study design and measures

The RHAPSODY and STEREO studies were conducted in accordance with the principles of the Declaration of Helsinki, and the protocols were approved by the institutional review boards of the participating centers. All patients provided written informed consent before any study-related procedures were initiated [18,19].

In both studies, patients subcutaneously self-administered adalimumab, 40 mg (Abbott Laboratories, Abbott Park, IL) every other week in addition to their preexisting antirheumatic treatments for a core study period of 12 weeks, with an optional extension period up to week 20. For patients who had prior exposure to TNF antagonists, study investigators documented the reasons for discontinuation of IFX and/or ETN in four prespecified categories: never achieved response (lack of response), loss of initial response (loss of response), adverse effects (intolerance), and other. Answers were not mutually exclusive. Evaluations of effectiveness and safety were conducted at weeks 2, 6, 12, and 20, as applicable.

Measures of effectiveness of adalimumab for patients with AS included ≥ 40% improvement in the Assessment of SpondyloArthritis International Society criteria (ASAS40) [22], ≥ 50% improvement in the BASDAI (BASDAI 50) [23], and changes in BASDAI and the Bath Ankylosing Spondylitis Functional Index (BASFI) [24], as measured on a 0 to 10 cm horizontal visual analogue scale (VAS). Changes in swollen-joint count (SJC, 0 to 44) and tender joint count (TJC, 0 to 46) were calculated for patients with at least one swollen peripheral joint at baseline. Change in Maastricht Ankylosing Spondylitis Enthesitis Score (MASES, 0 to 13) [25] was evaluated for patients with enthesitis (one or more inflamed enthesis assessed by MASES) at baseline. Serum concentrations of CRP (milligrams per deciliter) are shown only for baseline.

Effectiveness measures for patients with PsA included the modified Psoriatic Arthritis Response Criteria (mPsARC) [26]; ≥ 50% improvement in the American College of Rheumatology response criteria (ACR50) [27]; TJC (0 to 78 joints), SJC (0 to 76 joints), and changes in TJC and SJC; and the Health Assessment Questionnaire Disability Index (HAQ DI, 0 to 3) [28]. Psoriasis severity was assessed by using the Physician's Global Assessment (PGA) 7-point scale with end points of "Clear " and "Severe" [29]. Psoriatic nail dystrophy was evaluated by using the Nail Psoriasis Severity Index (NAPSI [0 to 80], fingernails only) in patients with a NAPSI score ≥ 1 at baseline [30].

In both trials, adverse events (AEs) were collected throughout the treatment of each patient and for 70 days (five serum half-lives) after the last adalimumab injection.

### Statistical analyses

All patients who received at least one adalimumab injection were included in the analyses. Observed data at week 12 were used for all analyses of effectiveness. Patients in both studies were stratified into two major subgroups: no prior TNF antagonist and one or more prior TNF antagonists (prior IFX or ETN or both). Patients with a history of anti-TNF treatment were also stratified based on whether they had been treated with IFX, ETN, or both, and according to whether the reason for discontinuation of the prior TNF antagonist had been lack of response, loss of response, or intolerance. Analyses pertaining to the reason for discontinuation included only those patients who had stopped their prior anti-TNF therapy exclusively for one of these reasons. Thus, patients with more than one reason for prior discontinuation were excluded from analysis, unless the only additional reason was "other."

Descriptive analyses were performed by calculating counts and percentages for qualitative data and by calculating means, standard deviations, medians, and first and third quartiles (for strata with *n *< 50, minimum-maximum) for quantitative data. Based on previous evaluations of predictors of response, the following continuous variables were considered possible confounders: age (per year), disease duration (per year), BASDAI (AS) or TJC (PsA), SJC (PsA), CRP, and BASFI (AS) or HAQ DI (PsA) [18,19]. Categoric variables (yes versus no) evaluated as possible confounders included male sex, HLA-B27 (AS), SJC >0 (AS), enthesitis (AS), PGA < "Clear" (PsA), NAPSI >0 (PsA), and ongoing treatment with NSAIDs (AS) or DMARDs (PsA) [18,19]. Comparisons of the end points ASAS 40 and BASDAI 50 in subsets of patients with AS defined by prior IFX or ETN therapy or both (yes versus no), by prior anti-TNF therapy used (IFX, ETN, or both), and by reason for discontinuation of prior TNF antagonist (lack of response, loss of response, or intolerance) were performed by using logistic regression unadjusted and adjusted for baseline differences in possible confounders. ACR50 and mPsARC outcomes were compared unadjusted and adjusted in subsets of patients with PsA defined by prior IFX or ETN therapy or both (yes versus no).

## Results

### Prior anti-TNF therapy

In total, 1,250 patients with AS were enrolled in the RHAPSODY study. Of these, 924 had no history of anti-TNF therapy, and 326 (26%) patients had been treated with at least one TNF antagonist (162 patients with IFX, 85 patients with ETN, and 79 patients with both IFX and ETN [not concurrently] (Table 1), including 18 patients with IFX as the last TNF antagonist). In total, 442 patients with PsA were enrolled in the STEREO study. Of these, 376 had received no prior anti-TNF therapy, and 66 (15%) patients had been treated with at least one TNF antagonist (18 patients with IFX, 34 patients with ETN, and 14 patients with both IFX and ETN (not concurrently) (Table 2), including one patient with IFX as the last TNF antagonist). The mean/median duration of prior anti-TNF therapy was 18/15 months for patients with AS, and 20/16 months for patients with PsA. The mean/median time from the last dose of the last TNF antagonist to the first injection of adalimumab was 6.3/2.7 months for patients with AS and 6.1/2.0 months for patients with PsA. The reason for discontinuation of the prior TNF antagonist was lack of response for 64 patients with AS and 14 patients with PsA, loss of response for 115 patients with AS and 29 patients with PsA, and intolerance for 56 patients with AS and nine patients with PsA (Tables 1 and 2).

**Table 1:** Baseline demographic and disease characteristics of patients with AS stratified by prior anti-TNF therapy

	Patients with AS (N = 1,250)							
			**Prior TNF antagonist(s)**			**Reason for discontinuation of prior TNF antagonist^a^**		
				
	**No prior ETN/IFX**	**Prior ETN and/or IFX**	**IFX only**	**ETN only**	**IFX and ETN**	**Lack of response**	**Loss of response**	**Intolerance**
**Characteristic**	**(n = 924)**	**(n = 326)**	**(n = 162)**	**(n = 85)**	**(n = 79)**	**(n = 64)**	**(n = 115)**	**(n = 56)**

Male (%)	71	72	78	59	72	75	66	68
Age (years; mean ± SD)	44 ± 12	44 ± 11	44 ± 10	44 ± 12	43 ± 10	45 ± 10	42 ± 10	44 ± 13
AS duration (years; mean ± SD)	11 ± 10	11 ± 9	12 ± 9	9 ± 9	11 ± 8	10 ± 9	11 ± 8	12 ± 10
HLA-B27 positive (%)	83	81	80	82	81	86	82	84
Peripheral arthritis (%)^b^	22	25	26	27	19	23	24	34
Enthesitis (%)^c^	55	55	52	57	60	50	56	64
Concomitant NSAIDs (%)	77	66	69	57	71	53	68	66
C-reactive protein (mg/dl)^d^	1.3	1.2	1.4	1.1	1.1	1.4	1.6	0.8
	0.6, 2.6	0.5, 2.6	0.5, 3.0	0.4, 2.2	0.4, 2.6	0.7, 2.8	0.6, 2.7	0.3, 1.7

^a^No additional reason except for "other." ^b^One or more swollen joint count (0 to 44). ^c^One or more inflamed enthesis in Maastricht Ankylosing Spondylitis Enthesitis Score or fascia plantaris or both. ^d^Reference value, 0.4 mg/dl; values listed are median and quartile 1, quartile 3 because of skewed distribution. AS, ankylosing spondylitis; ETN, etanercept; IFX, infliximab; NSAIDs, nonsteroidal antiinflammatory drugs; TNF, tumor necrosis factor.

**Table 2:** Baseline demographic and disease characteristics of patients with PsA stratified by prior anti-TNF therapy

	Patients with PsA (N = 442)							
			**Prior TNF antagonist(s)**			**Reason for discontinuation of prior TNF antagonist^a^**		
				
	**No prior ETN/IFX**	**Prior ETN and/or IFX**	**IFX only**	**ETN only**	**IFX and ETN**	**Lack of response**	**Loss of response**	**Intolerance**
**Characteristic**	**(n = 376)**	**(n = 66)**	**(n = 18)**	**(n = 34)**	**(n = 14)**	**(n = 14)**	**(n = 29)**	**(n = 9)**

Male (%)	50	47	61	41	43	50	45	44
Age (years; mean ± SD)	48 ± 11	47 ± 12	44 ± 12	47 ± 13	50 ± 8	43 ± 10	48 ± 13	51 ± 11
PsA duration (years; mean ± SD)	10 ± 8	12 ± 8	13 ± 7	9 ± 8	17 ± 8	7 ± 5	14 ± 7	14 ± 11
Psoriasis (%)^b^	83	80	78	79	86	79	79	78
Nail dystrophy (%)^c^	59	55	72	41	64	43	62	11
Concomitant DMARDs (%)	71	52	72	35	64	29	59	44
Concomitant glucocorticoids (%)	26	44	44	41	50	36	41	44
C-reactive protein (mg/dl)^d^	0.9	1.3	0.8	1.5	1.1	0.8	1.6	1.4
	0.4, 2.2	0.3, 2.8	0.1 to 8.4^e^	0.1 to 7.0^e^	0.5 to 4.7^e^	0.1 to 7.0^e^	0.1 to 8.4^e^	0.2 to 3.4^e^

^a^No additional reason except for "other." ^b^Patients with PGA other than "Clear." ^c^Patients with NAPSI score ≥ 1. ^d^Reference value, 0.4 mg/dl; values listed are median and quartile 1, quartile 3 because of skewed distribution. ^e^Ranges presented are minimum to maximum because of small numbers of patients per group. DMARDs, disease-modifying antirheumatic drug; ETN, etanercept; IFX, infliximab; NAPSI, nail psoriasis severity index; NSAID, nonsteroidal antiinflammatory drug; PGA, Physician's Global Assessment of psoriasis; PsA, psoriatic arthritis; TNF, tumor necrosis factor.

### Patient disposition and exposure to adalimumab

Overall, 97% of 1,250 patients with AS completed the 12-week treatment with adalimumab. During the complete study, 1% of patients with AS (10 of 924) without prior anti-TNF therapy and 3% of patients with AS (11 of 326) with prior anti-TNF therapy withdrew because of an unsatisfactory response to adalimumab. Forty-four (5%) of patients without prior anti-TNF therapy and 22 (7%) of patients with previous anti-TNF therapy discontinued adalimumab because of AEs.

In the PsA study, 94% of 442 enrolled patients continued adalimumab therapy through week 12. During the study, 1% of TNF antagonist-naïve patients with PsA (five of 376) and 2% of patients with PsA previously treated with TNF antagonists (one of 66) discontinued adalimumab because of an unsatisfactory response. Six percent of patients (24 of 376) without prior anti-TNF therapy and 3% of patients (two of 66) with prior anti-TNF therapy withdrew from the study because of AEs.

For both studies, the mean/median treatment period with adalimumab was 15/12 weeks for all patients enrolled.

### Baseline demographics and disease characteristics

In both studies, demographics and the distribution of clinical manifestations were comparable between patients with and without prior anti-TNF therapy (Tables 1 and 2). Patients with AS and a history of anti-TNF therapy had slightly greater disease activity and more physical-function disability than did TNF antagonist-naïve patients (Table 3). Patients with PsA with and without histories of anti-TNF therapy had similar median TJCs and SJCs at baseline, whereas the functional impairment measured by the HAQ DI was greater for patients with a history of anti-TNF therapy than for anti-TNF agent-naïve patients (Table 4).

**Table 3:** Effectiveness of 12-week adalimumab treatment in patients with AS stratified by prior anti-TNF therapy

Outcome measure	No prior ETN/IFX	Prior ETN and/or IFX
	(n = 924)	(n = 326)
BASDAI (0 to 10)		
Baseline	6.2	6.5
	5.2, 7.2	5.5, 7.6
Change, Baseline to week 12	-3.7	-2.5
	-5.1, -1.9	-4.2, -1.0
BASFI (0 to 10)		
Baseline	5.2	6.0
	3.5, 7.0	4.3, 7.5
Change, Baseline to week 12	-2.2	-1.3
	-3.9, -0.8	-2.8, -0.1
Patients with peripheral arthritis (SJC ≥ 1)	n = 201	n = 80
TJC (0 to 46)		
Baseline	4	6
	2, 11	3, 14
Change, Baseline to week 12	-3	-3
	-8, -1	-8, -1
SJC (0 to 44)		
Baseline	2	2
	1, 4	1, 4
Change, Baseline to week 12	-2	-1
	-4, -1	-2, -1
Patients with enthesitis (MASES ≥ 1)	n = 492	n = 175
MASES (0 to 13)		
Baseline	5	5
	2, 8	3, 9
Change, Baseline to week 12	-3	-2
	-5, -1	-5 , -1

Values are expressed as median and quartile 1, quartile 3. AS, ankylosing spondylitis; BASDAI, Bath Ankylosing Spondylitis Disease Activity Index; BASFI, Bath Ankylosing Spondylitis Functional Index; ETN, etanercept; IFX, infliximab; MASES, Maastricht Ankylosing Spondylitis Enthesitis Score; TJC, tender joint count; TNF, tumor necrosis factor; SJC, swollen joint count.

**Table 4:** Effectiveness of 12-week adalimumab treatment in patients with PsA stratified by prior anti-TNF therapy

Outcome measure	No prior ETN/IFX	Prior ETN or IFX or both
	(n = 376)	(n = 66)
TJC (0 to 78)		
Baseline	16	15
	10, 27	8, 25
Change, Baseline to week 12	-10	-10
	-17, -5	-16, -3
SJC (0 to 76)		
Baseline	9	7
	5, 13	5, 11
Change, Baseline to week 12	-7	-5
	-10, -3	-8, -3
HAQ DI (0 to 3)		
Baseline	1.25	1.44
	0.75, 1.63	1.00, 1.75
Change, Baseline to week 12	-0.50	-0.50
	-0.88, -0.13	-0.75, -0.25
Patients with nail psoriasis (NAPSI ≥ 1)	n = 223	n = 36
NAPSI (0 to 80)		
Baseline	14	18
	5, 30	10, 37
Change, Baseline to week 12	-6	-6
	-14, -2	-15, -1

Values are expressed as median and quartile 1, quartile 3. ETN, etanercept; HAQ DI, Health Assessment Questionnaire Disability Index; IFX, infliximab; NAPSI, Nail Psoriasis Severity Index (only fingernails); PsA, psoriatic arthritis; SJC, swollen joint count; TJC, tender joint count; TNF, tumor necrosis factor.

### Effectiveness

#### Patients with AS stratified by history of anti-TNF therapy

At week 12, disease activity and physical impairment were notably reduced for all patient groups, as indicated by BASDAI and BASFI; the changes were generally greatest for patients naïve to anti-TNF agents (Table 3). ASAS40 response rates were 59.3% for patients without prior TNF-antagonist treatment and 37.7% for patients with a history of anti-TNF therapy. BASDAI 50 responses were achieved by 63.0% of patients with AS without prior anti-TNF therapy and by 40.8% of patients with AS who had received prior anti-TNF therapy. Logistic regression showed that patients with prior anti-TNF therapy had a smaller likelihood of achieving an ASAS40 or BASDAI 50 response (Table 5).

**Table 5:** ASAS40 and BASDAI 50 response rates at week 12 in patients with AS stratified by prior anti-TNF therapy

	ASAS40			BASDAI 50		
		
	Response rate, *n/N *(%)	Unadjusted odds ratio	*P *value	Response rate, *n/N *(%)	Unadjusted odds ratio	*P *value
		(95% CI)			(95% CI)	
History of prior anti-TNF therapy						
Prior ETN or IFX or both	115/305 (37.7)	0.41 (0.32 to 0.54)	< 0.001	128/314 (40.8)	0.40 (0.31 to 0.53)	< 0.001
No prior ETN/IFX^a^	518/873 (59.3)			561/890 (63.0)		
Prior TNF antagonist(s)						
ETN only	25/81 (30.9)	0.93 (0.47 to 1.83)	0.083	27/81 (33.3)	0.98 (0.51 to 1.90)	0.033
IFX only	66/150 (44.0)	1.64 (0.91 to 2.93)		75/156 (48.1)	1.82 (1.03 to 3.20)	
ETN and IFX^a^	24/74 (32.4)			26/77 (33.8)		
Reason for discontinuation of prior TNF antagonist						
Loss of response	46/108 (42.6)	2.09 (1.05 to 4.14)	0.108	47/112 (42.0)	2.03 (1.03 to 4.03)	0.059
Intolerance	20/52 (38.5)	1.76 (0.79 to 3.91)		25/54 (46.3)	2.42 (1.11 to 5.30)	
Lack of response^a^	16/61 (26.2)			16/61 (26.2)		

^a^Used as reference value. AS, ankylosing spondylitis; ASAS40, Assessment of SpondyloArthritis international Society 40% response; BASDAI 50, Bath AS Disease Activity Index 50% response; CI, confidence interval; ETA, etanercept; IFX, infliximab; TNF, tumor necrosis factor.

ASAS40 and BASDAI 50 responses were greatest for the 162 patients with AS with only prior IFX therapy compared with patients with only prior ETN therapy and those with prior treatment with both IFX and ETN. In the logistic regression, the odds of achieving an ASAS40 response were not statistically different (*P *= 0.083) for patients with only prior IFX therapy or only ETN therapy, compared with the reference group of patients who had received both IFX and ETN (Table 5). In contrast, the odds of achieving a BASDAI 50 response were significantly greater for patients who had received only IFX (*P *= 0.033) (Table 5).

The observed ASAS40 and BASDAI 50 response rates at week 12 for patients stratified by reason for discontinuation of the prior TNF antagonist were greater for patients with loss of response or intolerance than for patients who had lack of response to the prior TNF antagonist (Table 5). However, unadjusted comparisons by logistic regression revealed that the probability of achieving ASAS 40 or BASDAI 50 responses was not statistically different for patients who discontinued prior anti-TNF therapy because of loss of response or intolerance compared with patients who experienced lack of response (Table 5). None of the results of the logistic regression analyses was relevantly changed after further adjustment for the baseline confounders listed previously.

Stratification first by exclusive treatment with IFX and then by reason for discontinuation resulted in observed BASDAI 50 response rates at week 12 of 48.5% for loss of response (n = 66), 52.6% for intolerance (n = 38), and 22.7% for lack of response (n = 22). For patients who had been treated only with ETN, the BASDAI 50 response rates at week 12 were 33.3% for loss of response (n = 33), 33.3% for intolerance (n = 12), and 32% for lack of response (n = 12). The pattern for ASAS40 response rates was overall similar for patients with previous ETN therapy. The ASAS40 response rates in patients who discontinued IFX stratified by reason for discontinuation showed smaller differences than the BASDAI 50 response rates: 50.8% for loss of response, 38.9% for intolerance, and 27.3% for lack of response.

At week 12 of the study, patients with AS with peripheral arthritis or with enthesitis at baseline and previous treatment with TNF antagonists experienced quite similar improvements in joint counts and in MASES, as did patients without a history of anti-TNF therapy (Table 3).

#### Patients with PsA stratified by history of anti-TNF therapy

The median changes in TJC, SJC, and HAQ DI were very similar for patients without and with previous TNF-antagonist treatment (Table 4). At week 12, the percentages of patients who achieved ACR50 and mPsARC responses were somewhat greater for those without a history of anti-TNF therapy than for those with prior anti-TNF therapy (ACR50: 52.3% versus 41.7%; mPsARC: 78.8% versus 71.2%). Unadjusted logistic regression revealed that the likelihood of attaining an ACR50 response or mPsARC response did not considerably differ between patients without and those with prior anti-TNF therapy (Table 6). The results remained unchanged after adjustment for other confounders at baseline.

**Table 6:** ACR50 and mPsARC response rates at week 12 in patients with PsA stratified by prior anti-TNF therapy

	ACR50			mPsARC		
		
	Response rate,	Unadjusted odds ratio	*P *value	Response rate,	Unadjusted odds ratio	*P *value
	*n/N *(%)	(95% CI)		*n/N *(%)	(95% CI)	
History of prior anti-TNF therapy						
Prior ETN or IFX or both	25/60 (41.7)	0.65 (0.37 to 1.13)	0.130	42/59 (71.2)	0.66 (0.36 to 1.23)	0.194
No prior ETN/IFX^a^	182/348 (52.3)			272/345 (78.8)		

^a^Used as reference value. ACR50, American College of Rheumatology 50% or more improvement; CI, confidence interval; ETN, etanercept; IFX, infliximab; mPsARC, modified Psoriatic Arthritis Response Criteria; PsA, psoriatic arthritis.

At week 12, ACR50 and mPsARC responses were achieved by 10 and 13, respectively, of 18 patients with prior IFX therapy; by 11 and 18 of 30 patients with prior ETN therapy; and by four and 11 of 13 patients previously treated with both IFX and ETN (observed data). ACR50 and mPsARC responses were achieved by five and seven, respectively, of 13 patients with PsA with lack of response to prior TNF-antagonist treatment; by 10 and 17 of 27 patients with loss of response; and by five and seven of eight patients who were intolerant of prior anti-TNF therapy (observed data).

**Figure 1:**
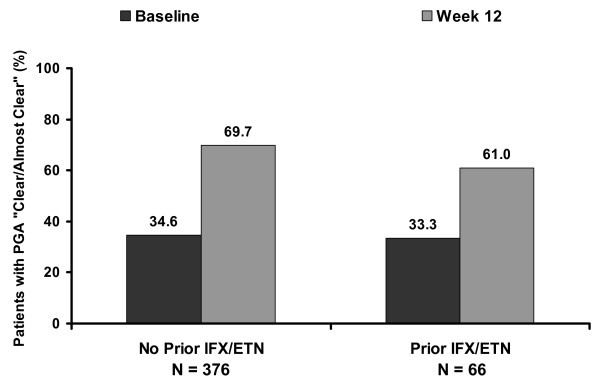
**Percentages of patients with psoriatic arthritis achieving a Physician's Global Assessment (PGA) of "Clear/Almost clear" at baseline and week 12, stratified by history of anti-tumor necrosis factor therapy**. Data are observed. IFX, infliximab; ETN, etanercept.

Patients achieving a PGA of "Clear/Almost clear" increased by 35.1 percentage points between baseline and week 12 for patients without prior TNF-antagonist treatment and by 27.7 percentage points for patients with a history of anti-TNF therapy (Figure 1). For patients with psoriatic nail dystrophy at baseline, the median change in NAPSI was similar in patients without and in patients with prior treatment with IFX and/or ETN (Table 4).

### Safety

The rates of serious adverse events (SAEs) were 3.1% and 4.3% for patients with AS without and with prior TNF-antagonist treatment, respectively. The rates of serious infections were 0.4% in patients with no prior exposure to TNF antagonists and 0.3% in patients who had received prior TNF-antagonist therapy. No serious allergic reaction was reported in the AS study.

For patients with PsA, the rates of SAEs were 4.3% and 3.0% in patients without and with prior TNF-antagonist therapy, respectively. Serious infections were documented for 0.5% of patients without prior anti-TNF therapy and for one patient (1.5%) with prior anti-TNF therapy. One TNF antagonist-naïve patient experienced a serious allergic reaction, which was the only one reported in the STEREO study. No cases of lupus, lupus-like reaction, or malignancies of any type were observed in either of the two studies.

## Discussion

This study represents the first evaluation of the effectiveness of adalimumab in patients with spondyloarthritis (AS and PsA) who had previously been treated with one or two other TNF antagonists. The disease characteristics of the patients in both studies closely matched those of patients considered eligible for anti-TNF therapy in daily practice [31,32].

After 12 weeks of adalimumab therapy, patients with AS or PsA having prior exposure to IFX or ETN or both experienced clinically important improvements of their diseases, as evaluated by standard outcome measures. However, response rates were generally lesser than those for patients who had not received prior anti-TNF therapy. Unadjusted and adjusted logistic regressions indicated that the likelihood of achieving BASDAI 50 and ASAS40 responses after 12 weeks was smaller for patients with than for those without a history of anti-TNF therapy. For patients with PsA, prior anti-TNF therapy also resulted in a lesser likelihood to achieve ACR50 (odds ratio, 0.65) and mPsARC (odds ratio, 0.66) responses, but this impact was not statistically important (ACR50 response rate, *P *= 0.130; mPsARC response rate, *P *= 0.194) (Table 6). In the PsA study, the impact of other confounders (sex, HAQ DI, joint counts, and PsA duration) was greater than that of prior anti-TNF therapy. Fewer patients were in the PsA study than in the AS study; therefore, the likelihood of detecting statistically important differences was less. The studies were not powered to detect differences between subgroups. However, assuming a response rate of 50% in one subgroup, an odds ratio of 1.5, and sample sizes of 900 versus 300 in the subgroups, the χ ^2 ^test would have >95% power to detect a difference in the response rates. The power would be 63% with sample sizes of 150 versus 80 and 61% with sample sizes of 350 versus 60.

Overall, the ASAS40 and BASDAI 50 response rates for patients with AS and a history of anti-TNF therapy were clinically meaningful (Table 5). Notably, adalimumab therapy reduced peripheral arthritis and enthesitis; the effectiveness of adalimumab for these extraaxial manifestations was very similar for patients with and without prior TNF-antagonist treatment (Table 3).

The ASAS40 and BASDAI 50 response rates were greater in patients who had been treated only with IFX therapy than in patients with only ETN therapy. However, the probability of achieving an ASAS40 response did not remarkably differ between patients with histories of treatment with IFX, ETN, or both. The likelihood of achieving a BASDAI 50 response was significantly greater for patients with prior IFX therapy.

Logistic regression analyses demonstrated that the reason for discontinuation of the prior TNF antagonist had no statistically important impact on the chances of experiencing ASAS40 or BASDAI 50 responses. However, the observed ASAS40 and BASDAI 50 response rates were greater for patients who discontinued prior anti-TNF therapy because of loss of response or intolerance than they were for those patients who discontinued the first anti-TNF agent because of lack of response. Stratifications first by type of prior TNF antagonist and then by reason for discontinuation showed similar response rates to adalimumab in patients who had lack of response to the preceding TNF antagonist. Patients who had initially responded to prior IFX and discontinued IFX because of loss of response (perhaps caused by antibodies against IFX) or because of intolerance experienced much greater BASDAI 50 and ASAS40 response rates than did patients who discontinued IFX after lack of initial response. In patients who had been treated with only ETN, stratification by reason for discontinuation showed overall similar response rates across the subsets. The interpretation of this result is limited by the small numbers of patients who had been treated only with ETN. Thus, patients who initially respond to a first TNF antagonist may be more likely also to respond to a second TNF antagonist, in particular when the type of agent is similar (monoclonal antibody). These results are generally consistent with previous analyses of patients with RA treated with adalimumab as the second or third TNF antagonist in the ReAct trial (Research in Active Rheumatoid Arthritis Trial) [5]. ReAct, STEREO, and RHAPSODY were all designed to follow routine clinical practice for patient enrollment.

Patients with PsA who had prior IFX or ETN therapy or both achieved clinically meaningful ACR50 and mPsARC response rates of 41.7%, and 71.2%, respectively. The history of anti-TNF therapy had no relevant impact on the likelihood of achieving an ACR50 response in the unadjusted evaluations (as mentioned earlier, the adjustment for confounders did not change this result). Patients with PsA who had lack of response to prior TNF-antagonist therapy experienced somewhat less clinical improvement with adalimumab compared with patients with loss of response or intolerance to their prior TNF-antagonist treatment. Adalimumab was similarly effective for patients with prior IFX or ETN therapy. However, no logistic regression was performed owing to small group sizes. Adalimumab was also highly effective for psoriatic skin and nail lesions for patients with a history of anti-TNF therapy, with improvements similar to those of patients with PsA who were naive to TNF antagonists.

A limitation of these evaluations is the open-label study design and the explorative nature of the investigations. The response rates in patients without prior anti-TNF therapy were generally greater than the rates reported in randomized clinical trials of adalimumab [33,34], ETN [35,36], and IFX [37,38] in AS or in PsA, in which prior treatment with other TNF antagonists was generally prohibited. For example, the ASAS40 response at week 12 was 59.3% in patients with AS without prior anti-TNF therapy in this open-label study and 39.9% in the adalimumab ATLAS trial [33]. In patients with PsA, the ACR50 response rate was 52.3% for patients without prior anti-TNF therapy in this open-label study and 36% in the adalimumab ADEPT trial [34]. A potential explanation for the greater response rates in our open-label, explorative studies is that the evaluations were based on observed data and omission of missing data, whereas patients with missing data were imputed to be nonresponders in the intention-to-treat analyses of randomized trial data. Furthermore, patients (and physicians) may somewhat overestimate the treatment effect in open-label studies, particularly when no alternative treatment is available, as is often the case for patients with AS.

## Conclusions

Patients with AS and patients with PsA previously treated with TNF antagonists experienced clinically important improvements in their diseases after 12 weeks of adalimumab therapy during these two large, open-label clinical studies. The safety profile of adalimumab was similar in patients with and without prior anti-TNF therapy and consistent with results from other adalimumab clinical trials.

## Abbreviations

ACR: American College of Rheumatology; AS: ankylosing spondylitis; ASAS: Assessment of SpondyloArthritis International Society; BASDAI: Bath Ankylosing Spondylitis Disease Activity Index; BASFI: Bath Ankylosing Spondylitis Functional Index; CRP: C-reactive protein; DMARDs: disease modifying antirheumatic drugs; ETN: etanercept; HAQ DI: Health Assessment Questionnaire Disability Index; IFX: infliximab; MASES: Maastricht Ankylosing Spondylitis Enthesitis Score; mPsARC: modified Psoriatic Arthritis Response Criteria; NAPSI: Nail Psoriasis Severity Index; NSAIDs: nonsteroidal antiinflammatory drugs; PGA: Physician's Global Assessment; PsA: psoriatic arthritis; RA: rheumatoid arthritis; SJC: swollen joint count; TJC: tender joint count; TNF: tumor necrosis factor; VAS: visual analogue scale.

## Competing interests

MR has served as a consultant and has received speaking fees and honoraria from Abbott, MSD, Schering-Plough, Pfizer, and Wyeth. FVdB has received speakers bureau honoraria and/or has served as a consultant for Abbott Laboratories, Schering-Plough, UCB, and Wyeth. MK and HK are full-time employees of Abbott GmbH & Co. KG, an affiliate of Abbott Laboratories and hold shares of Abbott stock. SK is a contractor of Abbott GmbH & Co. KG.

## Authors' contributions

MK and HK (with other academic experts and members of Abbott Laboratories) designed the RHAPSODY and STEREO clinical trials. MR was the principal investigator for the RHAPSODY study. FVdB was the principal investigator for the STEREO study. MK designed and performed the statistical analyses. SK drafted the manuscript in cooperation with the principal investigators and coauthors. All authors reviewed and approved the final content of the submitted manuscript.
